# In Vivo Super-Resolution Cardiac Diffusion Tensor MRI: A Feasibility Study

**DOI:** 10.3390/diagnostics12040877

**Published:** 2022-03-31

**Authors:** Anne-Lise Le Bars, Kevin Moulin, Daniel B. Ennis, Jacques Felblinger, Bailiang Chen, Freddy Odille

**Affiliations:** 1 IADI, Inserm U1254, Université de Lorraine, 54000 Nancy, France; annelise.lebars@gmail.com (A.-L.L.B.); j.felblinger@chru-nancy.fr (J.F.); 2 Department of Radiology, Stanford University, Stanford, CA 94305, USA; kevin.moulin.26@gmail.com (K.M.); dbe@stanford.edu (D.B.E.); 3 CIC-IT 1433, Inserm, CHRU de Nancy, Université de Lorraine, 54000 Nancy, France; b.chen@chru-nancy.fr

**Keywords:** cardiac magnetic resonance imaging, diffusion tensor imaging, super-resolution reconstruction, motion correction

## Abstract

A super-resolution (SR) technique is proposed for imaging myocardial fiber architecture with cardiac magnetic resonance. Images were acquired with a motion-compensated cardiac diffusion tensor imaging (cDTI) sequence. The heart left ventricle was covered with three stacks of thick slices, in short axis, horizontal and vertical long axes orientations, respectively. The three low-resolution stacks (2 × 2 × 8 mm^3^) were combined into an isotropic volume (2 × 2 × 2 mm^3^) by a super-resolution reconstruction. For in vivo measurements, each slice was acquired during a breath-hold period. Bulk motion was corrected by optimizing a similarity metric between intensity profiles from all intersecting slices in the dataset. The benefit of the proposed approach was evaluated using a numerical heart phantom, a physical helicoidal phantom with artificial fibers, and six healthy subjects. The SR technique showed improved results compared to the native scans, in terms of image quality and cDTI metrics. In particular, the myocardial helix angle (HA) was more accurately estimated in the physical phantom (HA = 41.5° ± 1.1°, with the ground truth being 42.0°). In vivo, it resulted in a sharper rate of change of HA across the myocardial wall (−0.993°/% ± 0.007°/% against −0.873°/% ± 0.010°/%).

## 1. Introduction

Diffusion magnetic resonance imaging (MRI) is a non-invasive imaging modality that allows the assessment of microarchitecture in biological tissues. In particular, cardiac diffusion tensor imaging (cDTI) reveals the three-dimensional myocardial microstructure and myofiber orientation. Knowing the arrangement of cardiomyocytes is important to understand cardiac electrical conduction and contractility [1,2,3]. Alterations of the microstructure have been observed in various cardiomyopathies including myocardial infarction and heart failure [4,5,6,7]. The microstructure can be described by the mean diffusivity (MD) and the fractional anisotropy (FA), which are derived from the diffusion tensor [8]. The first eigenvector $e_{1}$ of the diffusion tensor provides the direction of the longitudinal axis of the cardiomyocytes and the second eigenvector $e_{2}$ lies in the sheetlet/shear plane [4,9]. The first eigenvector direction is described by the helix angle (HA) and the transverse angle (TA), which are defined in a local cardiac coordinate system [10].

The main challenge of in vivo cDTI is to handle patient motion, including cardiac contraction and breathing. To deal with cardiac motion, a cardiac-triggered diffusion-weighted spin-echo echo planar imaging (DW SE-EPI) sequence can be used with first and second order motion-compensated diffusion-encoding gradients [11,12,13,14,15]. Breathing motion can be dealt with by repeated breath-holding or free-breathing with echo-navigation [16]. The cDTI data acquisition is a relatively slow imaging technique as several diffusion-encoding directions (at least six) and multiple averages are required in order to obtain sufficient signal-to-noise ratio (SNR) and accuracy. As an example, using such a sequence at 1.5 T as in [17], with b = 450 s/mm^2^, 12 directions, 16 averages, navigator gating, the authors achieved a spatial resolution of 2.8 × 2.8 mm^2^ (single-slice of 8 mm thickness) in 7 min on average, with good inter-session reproducibility. Consequently, due to long acquisition times, human in vivo cDTI has mostly been performed on a limited number of slices (typically one to three) in short-axis (SAX) orientation. Nevertheless, whole-heart coverage is possible and has been successfully performed with a limited resolution in the slice direction, typically 8 mm [10,18,19]. Acceleration methods such as simultaneous multi-slice imaging have been proposed [20] and applied to obtain a clinically feasible whole-heart coverage. This enabled 3D reconstruction of the myocardial fibers using tractography, but with non-isotropic voxels [18]. Note that isotropic voxels are recommended to apply such tractography algorithms [21].

To address the problem of resolution, several super-resolution methods have been explored for DTI and successfully applied to ex vivo cDTI [22,23,24]. Super-resolution can be achieved by combining multiple stacks of images acquired with different orientations. The images to be combined need to be bulk motion-corrected to avoid blurring when the organs have moved between the acquisitions. The multiple stack strategy has potentially several advantages: it may be beneficial for SNR and for averaging out EPI artifacts, but also for motion correction. Indeed, line profiles at the intersection of image planes from different stacks should be consistent. Therefore, the motion problem can be solved by aligning imaging planes in a way that best matches all their intersections. Such a registration method has been applied successfully to reconstruct 3D volumes of the human fetal brain [25].

In this work we propose to adapt this bulk motion-corrected super-resolution approach to cDTI. Repeated breath-holds are performed to acquire three stacks of slices, in SAX, horizontal long axis (HLAX) and vertical long axis (VLAX) orientations. Motion-compensation was performed: (i) prospectively, using a DW SE-EPI sequence with first and second order motion-compensated diffusion gradients; and (ii) retrospectively, using a slice-to-stack rigid registration technique enforcing self-consistency of slice intersections. The goal of this study is to demonstrate the feasibility of super-resolution in vivo cDTI using the proposed approach. Image quality, SNR and diffusion tensor estimation were evaluated in a numerical heart phantom, in a physical helicoidal phantom, and in vivo in volunteers by comparison with whole ventricle coverage using thick short-axis slices.

## 2. Materials and Methods

### 2.1. Super-Resolution Reconstruction

**Figure 1:**
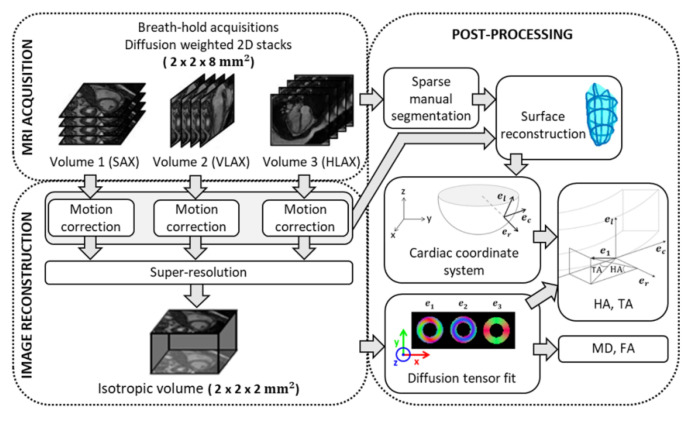
Acquisition, reconstruction and post-processing pipeline for 3D diffusion in vivo cardiac imaging. SAX: short axis; VLAX: vertical long axis; HLAX: horizontal long axis; $e_{1},\;e_{2},\;e_{3}$: eigenvectors of diffusion tensor; $e_{r},\;e_{c},\;e_{l}$: radial, circumferential and longitudinal axis of the local cardiac coordinate system; MD: mean diffusivity; FA: fractional anisotropy; HA: helix angle; TA: transverse angle.

The problem of reconstructing a 3D isotropic volume (i.e., a 3D volume with isotropic voxels), from multiple 3D or 2D multi-slice datasets with anisotropic voxels has been termed as super-resolution not only in the MRI literature [26,27] but also in other fields such as remote sensing [28]. The super-resolution reconstruction used here has been described before to retrieve a high-resolution isotropic volume from three stacks of images with thick slices [29]. It is illustrated in Figure 1. In this work, the three stacks correspond to the SAX, HLAX and VLAX of the heart and are not strictly orthogonal. In the forward problem, the stack of images for the $i^{th}$ orientation are modeled by a rotation operator and a slice selection operator applied to the isotropic volume. Therefore, the optimization problem to be solved can be written as:

(1) $$\underset{\rho_{iso}}{\mathrm{argmin}}\sum_{i=1}^{N}∥D_{i}B_{i}T_{i}\rho_{iso}-\rho_{i}∥{}^{2}+\lambda Q\left(\rho_{iso}\right),$$

where $T_{i}$ is the rotation between the reconstructed isotropic volume and the $i^{th}$ orientation, $D_{i}$ and $B_{i}$ are, respectively, a down-sampling and a blurring operator in the slice direction, $\rho_{iso}$ is the high-resolution isotropic volume and $\rho_{i}$ is the stack of images in the $i^{th}$ orientation. The regularizer for the ill-conditioned problem is chosen to be the Beltrami energy, which is a variant of the total variation energy: $Q\left(\rho_{iso}\right)={\left(1+\beta^{2}{\left|\nabla \rho_{iso}\right|}^{2}\right)}^{1/2}$ where β is the Beltrami constant. This regularization allows the preservation of edge sharpness and a reduction in noise, as well as reducing the stair-casing effects [30]. The choice of the Beltrami constant (β=1) and the regularization parameter $\lambda =10^{-5}$ are based on previous studies where they were shown to provide a good quality of reconstruction [29,31]. As demonstrated previously, in diffusion MRI, a super-resolution strategy is more efficient than a native high-resolution scan because it increases the number of excited spins per unit of time, resulting in a substantial improvement in SNR [31].

### 2.2. Numerical Simulations

#### 2.2.1. Numerical Phantom

To evaluate the super-resolution reconstruction strategy for cDTI measurements, a simplified model of the left ventricle (LV) was implemented in MATLAB (The MathWorks, Natick, MA, USA). A synthetic semi-ellipsoid object was created within a 160 × 160 × 128 image, with the following equation:

(2) $${\left(\frac{x-x_{c}}{R_{min}+dr}\right)}^{2}+{\left(\frac{y-y_{c}}{R_{min}+dr}\right)}^{2}+{\left(\frac{z-z_{c}}{R_{max}+dr}\right)}^{2}=1,$$

where $\left(x_{c},y_{c},z_{c}\right)$ is the center of the ellipsoid, $R_{min}=16$, $R_{max}=60$ and dr∈[0,14] is the distance to the endocardium. A local cardiac coordinate is assigned to each voxel that belongs to the object. To model the global structure of cardiomyocytes in a healthy heart, HA was chosen to vary linearly from −84° at the epicardium border to 84° at the endocardium border and TA was set to 0° [4,9]. The diffusion tensor was generated from its eigenvectors and eigenvalues (we chose $\lambda_{1}=2\times 10^{-3\;}$ mm^2^/s, $\lambda_{2}=1.5\times 10^{-3\;}$ mm^2^/s, $\lambda_{3}=1\times 10^{-3\;}$ mm^2^/s). Then, using the diffusion tensor model, D, the DW signal was given by $S\left(n\right)=S_{0}e^{-bg_{n}Dg_{n}^{T}}$ where S(n) is the signal obtained with the gradient direction $g_{n}$ and $S_{0}$ is the signal without diffusion weighting. Finally, we obtained a set of synthetic DWI volumes with one non-diffusion weighted image (b=0 s·mm^−2^) and six diffusion directions that follow a dual gradient scheme [32] and have a b-value of 350 s·mm^−2^. This set of images with a voxel size of 1 × 1 × 1 mm^3^ serves as the ground truth (GT).

The reduction of spatial resolution in frequency and phase directions was achieved by cropping the k-space by a factor of two. The slice selection was modelled as an ideal rectangular function. Eventually, four sets of DWI were generated with a voxel size of 2 × 2 × 2 mm^3^ for native high-resolution images (HR) and 2 × 2 × 8 mm^3^ for low resolution images (LR). The frequency/phase plane was chosen to lie in the xy plane for the first LR set, in the yz plane for the second LR set and in the xz plane for the third LR set. Rician noise was added to all images. The SNR of LR images was four times that of HR images because of the larger voxel size. Super-resolution (SR) volumes were reconstructed from the LR images and had an isotropic resolution equal to that of the HR resolution. Finally, LR, HR and SR images were interpolated to match the resolution of ground truth images.

#### 2.2.2. Data Analysis

Three different acquisition strategies were simulated and evaluated, which would all correspond to the same scan time for the corresponding same slice coverage: direct acquisition of HR DWI, acquisition of LR DWI in the xy plane with three repetitions and acquisition of sets of LR DWI in three orthogonal orientations for SR reconstruction. All images were resampled to 1 × 1 × 1 mm^3^ for visualization and comparative analysis to the GT data.

Analysis of the DTI reconstruction was completed on a slightly eroded region of the ventricle in order to suppress the partial volume effects that occur at the edge of the heart phantom. HA measures were compared to the GT using the mean absolute error and Bland–Altman plots. Differences with the GT were also analyzed for MD, FA and TA, which are expected to be uniform inside the heart region.

### 2.3. Physical Phantom

#### 2.3.1. Setup and Data Acquisition

To validate the super-resolution reconstruction and to evaluate the influence of slice orientation on cDTI measurements such as helix angle, we used a home-made phantom.

**Figure 2:**
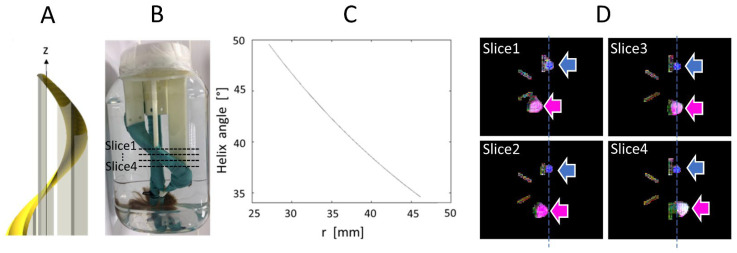
(**A**). Helicoidal holder for the fiber bundle; (**B**). Picture of the object; (**C**). Variation of helix angle in function of the distance, r, to the z axis which intersect the center of the support; (**D**). Ground truth MR images of the phantom (four axial slices from top to bottom, approximate slice locations are indicated in (**B**)), shown as color-coded FA maps, where the pink and blue arrows indicate the straight and helical bundles, respectively.

The phantom was composed of a bundle of artificial fibers attached to a 3D-printed helicoidal plastic holder, as shown in Figure 2. The holder is a structure that rotates by 360° about the z axis, with an elevation of 200 mm. The holder has a minimal radius of 19.08 mm and a maximal radius of 27.12 mm. Therefore, the helix angle varies as a function of radius from 34.57° to 49.57° and the mean angle is 42.07°. These values serve as ground truth for our HA measurements. Each single fiber is a composite of acrylic-polyester, similar to synthetic hair, and has a diameter of approximately 100 μm. The bundle was held tight by a wrapping of plastic-coated fabric. The phantom was immersed in water.

DWI were acquired on a clinical scanner (3T, MAGNETOM Prisma, Siemens, Erlangen, Germany) equipped with an 80 mT/m and 200 T/m/s slew rate gradient coil system. An 18-channel cardiac coil array was used in combination with a 32-channel spine coil array. The phantom was positioned so that its z axis aligned with the z axis of the scanner. Residual misalignment was corrected in post-processing using fixed landmarks. The sequence used for data acquisition was a standard DW SE-EPI with monopolar diffusion-encoding gradients. The imaging parameters were: TE/TR = 45 ms/2000 ms, GRAPPA factor 2, partial Fourier factor 6/8, b = 0, 500 s/mm^2^, 200 mm field-of-view (FOV), matrix size 100 × 80 (phase FOV 80%), no interpolation. To validate HA measurements, we first acquired a high-resolution image with a voxel size of 2 × 2 × 2 mm^3^, 20 slices, 30 diffusion directions and 20 repetitions. The use of partial Fourier and parallel imaging acceleration (GRAPPA), in combination with a moderate in-plane resolution (2 mm), resulted in the EPI readout remaining relatively short, thus minimizing geometric distortions. Another set of high-resolution images was acquired with 6 diffusion directions and 60 repetitions, using the same gradient direction scheme as in the in vivo experiments (dual gradient scheme, i.e., directions [1 0 −1], [1 0 1], [0 −1 −1], [0 −1 1], [1 −1 0], [1 1 0]) and resulting in the same acquisition time as the SR scan. The SR scan consisted of three sets of low-resolution images with a voxel size of 2 × 2 × 8 mm^3^ in transverse, coronal and sagittal orientation, each of them using the same 6-direction “dual gradient” scheme. For comparison, all images were reformatted to the same orientation and resolution as the high-resolution.

#### 2.3.2. Statistical Analysis

A circular region of interest (ROI) was positioned inside the fiber bundle for each of the 20 slices. The radius of each circular ROI was 8 mm, chosen to best fit the entire helical bundle section on each slice. By construction of the phantom, HA is expected to be constant across the slices. However, there is a slight variation of HA in the radial direction, as shown by the graph in Figure 2C. Since this radial variation is small, we computed the mean, minimum and maximum of HA for each slice, and we reported statistics of these metrics across the 20 slices (mean and standard deviation). These values were compared with the ground truth theoretical value, which was used to design the phantom. TA was computed in the same way. Note that this phantom design (see Figure 2A,B) is aimed at evaluating HA, which is expected to be more consistent across the helical holder. TA was also computed; however, the manual tightening of the artificial bundles did not ensure that TA was consistent throughout the bundle. SNR was measured on the non-diffusion-weighted image by the average signal in the bundle ROI divided by the standard deviation of the signal in a ROI placed in the background.

### 2.4. In Vivo Data

#### 2.4.1. Data Acquisition

The super-resolution acquisition strategy was applied to six healthy subjects (5 males and 1 female, age range 25–56 years old, weight range 53–81 kg) who had a mean heartrate of 59 ± 12 bpm. The volunteer study was approved by an ethics committee and informed written consent was obtained (ClinicalTrials.gov identifier: NCT02887053). DWI was performed with a first and second order motion-compensated DW SE-EPI sequence [11]. This motion compensation provides a good trade-off between signal loss due to a longer diffusion gradient (and thereby a longer TE) and motion sensitivity of the sequence. To reduce the length of the single-shot EPI readout we used parallel imaging with a GRAPPA factor of 2, inner-volume excitation and a partial Fourier. The sequence parameters were: 256 mm FOV, matrix size 128 × 104 (phase FOV 81%), no interpolation, in-plane resolution = 2 × 2 mm^2^, slice thickness = 8 mm, TE = 54 ms, TR = 1 RR, b = 0 s/mm^2^ plus six diffusion encoding directions with b = 350 s/mm^2^ and two repetitions. A reduced FOV technique was used to image a small FOV focused on the heart, without aliasing. For one volunteer who had a very low heart rate (42 bpm), the b value was set to 500 s/mm^2^. The acquisitions are ECG triggered with a trigger delay equal to the mid-systolic time point [12]. This cardiac phase is recommended with the second order motion compensated sequence because it corresponds to a “sweep spot” where cardiac motion is more regular, making diffusion measurements more robust to cardiac motion. The set of DWI corresponding to one slice was acquired within a breath-hold of approximately 17 s (17 heart beats). To perform the super-resolution reconstruction, three stacks of images were acquired with the same imaging parameters in SAX, HLAX and VLAX orientations (see Figure 1). Typically, 15, 12 and 12 slices are required to cover the entire ventricle in the SAX, HLAX and VLAX orientations, respectively. So, a total scan time for the three stacks, including rest periods between breath-holds, was approximately 18 min.

#### 2.4.2. Motion Correction Strategy

**Figure 3:**
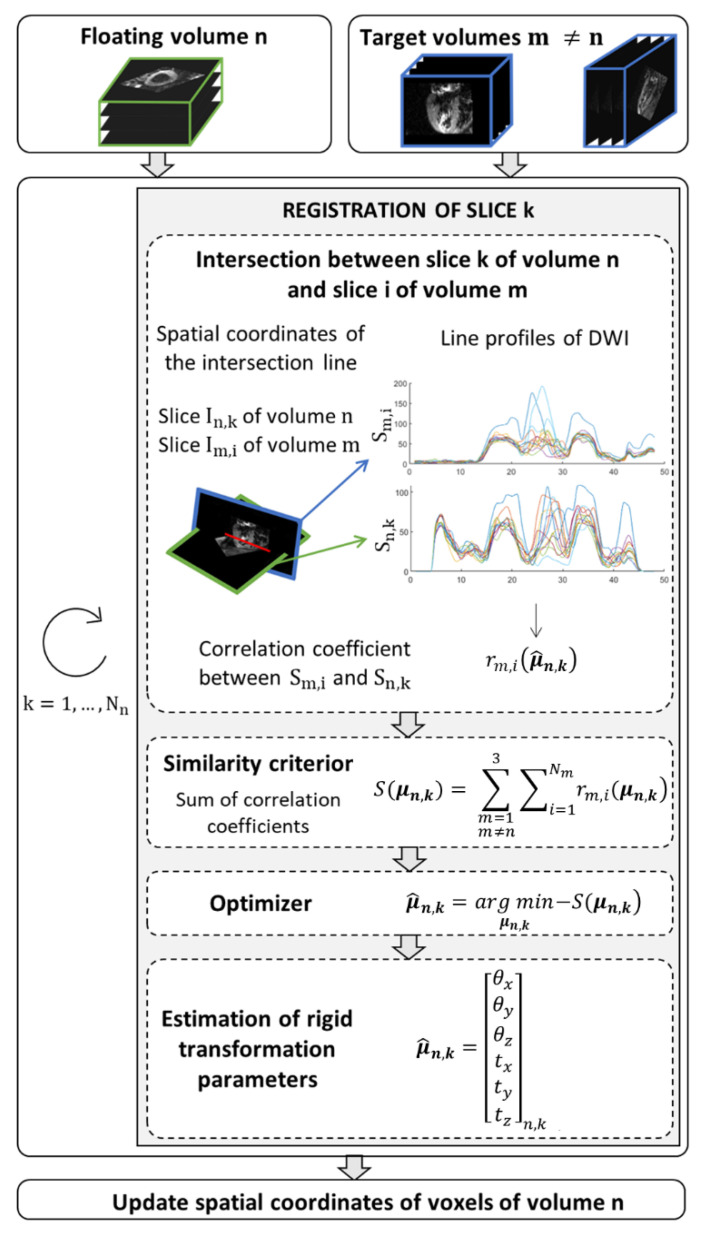
Algorithm to find the new spatial coordinates of voxels that ensure a global consistency between the floating volume *n* and the other two volumes (target) in the domain shared by the three orientations. The similarity criterion, *S*, corresponds to the summation of correlation coefficients $r_{m,i}$($\mu_{n,k}$) between diffusion weighted images $I_{n,k}$ extracted of the volume *n* and reference diffusion weighted images, $I_{m,i}$. The intensity level line profiles interpolate at points that belong to the intersection of the two image planes that serve to calculate the correlation coefficients. The parameters vector ${\hat{\mu }}_{n,k}$ of rigid transformation to apply to the slice *k* of volume *n* is estimated by minimizing the similarity criterion.

Before applying SR reconstruction to in vivo data, a pre-processing step was applied to correct for motion due to inconsistent breath-holding from slice to slice. The proposed method is a slice-to-stack registration, adapted from previous work in fetal imaging [25]. It consists of applying the rigid transformation (three translation and three rotation parameters) that best aligns a given slice to the intersecting slices in other stacks. The spatial position of each slice is determined in the patient coordinate system. The SR reconstruction is applied to the intersection volume shared by the three orientations, $\Omega \subset {\mathbb{R}}^{3}$. To find the optimal rigid transformation parameters for a given slice, we first calculate the equation of intersection lines between a given slice *k* in stack *n* (called the floating slice) and all slices in other stacks (called target slices). The registration of the floating slice consists of searching for the rigid transformation of the imaging plane that best matches the intensity profiles at those line intersections within the reconstruction volume Ω. The optimization problem to solve is:

(3) $${\hat{\mu }}_{n,k}=\underset{\mu_{n,k}}{\mathrm{argmin}}-S\left(\mu_{n,k}\right),$$

where $\mu_{n,k}$ is the vector of six components describing the rotation and translation of the rigid transformation. The similarity metric to be optimized, *S*, is based on the correlation coefficient and is expressed as:

(4) $$S\left(\mu_{n,k}\right)=\sum_{\begin{array}{c} m=1\\ m\neq n \end{array}}^{3}\sum_{i=1}^{N_{m}}r_{m,i}\left(\mu_{n,k}\right),$$

where $N_{m}$ is the number of slices in stack *m*. The correlation coefficient $r_{m,i}$ measures the similarity between the line profiles obtained from $I_{n,k}$, the DW images of the floating slice *k* in stack *n*, and $I_{m,i}$, the DW images of the target slice *i* in stack *m*. Optimization (3) is performed sequentially: one slice of a given stack is chosen as the floating slice, while slices in the other two stacks are considered as the target slices. This process, detailed in Figure 3, is repeated by changing the floating slice to the next slice in the stack, and so on until all slices from the stack have been optimized. Then the resulting rigid transformation is applied to the stack *n* and the other stacks are registered in the same way. An outer iteration loop is used to favor convergence of the overall optimization process.

As for implementation details, the number of outer iterations was set to five, an interior-point optimizer was used to solve each slice-to-stack registration subproblem (3), limit values were imposed on the search parameters (rotation angles < 5° and translations < 8 mm), and linear interpolations were used to calculate DWI profiles at intersection lines.

#### 2.4.3. Post-Processing

In order to calculate the descriptors of myofiber architecture (HA and TA), an accurate, subject-specific definition of the LV normal and LV tangent plane is needed. To compute local cardiac coordinates, a 3D mesh of the LV cavity was first constructed for each healthy volunteer. The 3D mesh was computed from manually segmented contours of the LV cavity. Given the high number of frames that compose the high-resolution image, a sparse segmentation strategy was employed for LV surface reconstruction as proposed in [29]. A total of five contours were segmented in the low-resolution images, including one HLAX and one VLAX slice that both intersected the apex, and three SAX slices (near base, mid-cavity and apex). Then, the contours were registered using the previously estimated rigid transformation parameters. This ensured a 3D consistency between all contours by correcting for motion that occurs between breath-holds, even for the low-resolution scans. This choice was made to make a fair comparison between SR and LR images, i.e., using optimal motion correction in all cases. Finally, the LV surface was computed by an implicit B-spline surface reconstruction algorithm which imposes a local smoothness constraints and is robust to large missing data [33]. The definitions used to calculate HA and TA from the cDTI eigenvectors and from the LV mesh are illustrated in Figure 1 and are similar to those described in [10].

#### 2.4.4. Statistical Analysis

Statistical analysis of the various cDTI metrics was conducted on five slices of the reconstructed volumes. Endocardial and epicardial contours were therefore segmented from these five slices in the non-diffusion weighted volumes. The five slices included three SAX slices (corresponding to XY planes) equally distributed between base and apex; the other two slices were chosen to be orthogonal to the SAX plane and to intersect the apex (corresponding to the XY and YZ planes). Note that the XZ and YZ orientations do not match HLAX and VLAX views. The LV wall region was divided into five layers from endocardium to epicardium to obtain the transmural profile. Next, the HA, TA, MD and FA measurements belonging to each layer were pooled for all healthy subjects in order to perform a global analysis of the performance of the reconstruction algorithm. A comparative ROI-based SNR analysis was performed using the ratio between the SNR measured on the SR reconstructed volume and the SNR measured on each native stack (after interpolation to the same voxel resolution). SNR was measured on the non-diffusion-weighted image by the average signal in the myocardium ROI divided by the standard deviation of the signal in a background ROI placed in the lung region.

## 3. Results

### 3.1. Numerical Phantom

**Figure 4:**
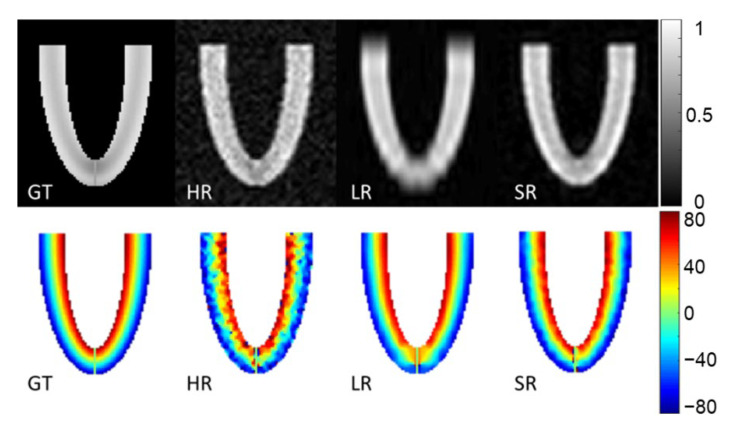
Magnitude image and helix angle (HA) reconstruction of the numerical phantom for a slice in XZ orientation. Ground truth (GT), high-resolution (HR), low-resolution (LR) and super-resolution (SR) magnitude images are in the first row (arbitrary units). The second row shows HA (degrees), ground truth (GT) and HA calculated from high-resolution (HR), low-resolution (LR) oriented in the XY plane and super-resolution (SR) datasets.

Figure 4 shows magnitude images of the three stacks of GT, HR, LR images and the SR reconstruction in the XZ plane. The second row of Figure 4 shows the HA ground truth and the reconstruction obtained with the three simulated acquisitions. The HR images look more affected by noise, which also appears in the HA maps. The LR magnitude images appear blurred, especially at the apex, although the impact on HA maps seems moderate. The SR images provide the highest fidelity in magnitude images compared to the GT. HA maps from LR and SR look similar and are both in good agreement with the GT.

**Figure 5:**
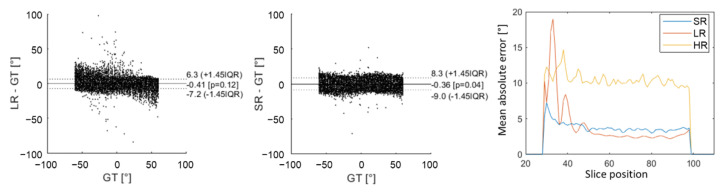
Comparison of HA obtained in the numerical phantom with low-resolution (LR) DWI and super-resolution (SR) to the ground truth (GT) using Bland–Altman graphs. The last graph on the right shows the evolution of mean absolute error as a function of slice position from apex to base. The yellow curve corresponds to high-resolution (HR) DWI, the red curve to LR DWI and blue curve to SR DWI.

Figure 5 shows an underestimation of high HA values with LR images, which arose from higher errors for slices no. 28 to 42 (near the apex). This effect appears on Bland–Altman graphs where there was a higher dispersion of HA errors with the LR dataset than with the SR dataset in comparison to ground truth. Nevertheless, the global interquartile range was higher with SR reconstruction because there were more errors for slices above 42 (mid-cavity to base). Overall, the mean absolute error was, respectively, 10.1°, 3.1° and 3.5° for HR, LR and SR images. Detailed mean MD, FA and TA mean values can be found in the Supplementary Materials (Table S1). The mean MD values were slightly underestimated for LR ((1.45 ± 0.02) × $10^{-3}$ mm^2^/s) in comparison to GT (1.50 ± 0.06) and SR (1.50  ± 0.02) that are not biased. For all of the metrics evaluated (MD, FA and TA), there were more errors with the HR dataset than with the other two. Values were all in good agreement with the GT, with a higher variability for HR datasets.

### 3.2. Physical Phantom

**Figure 6:**
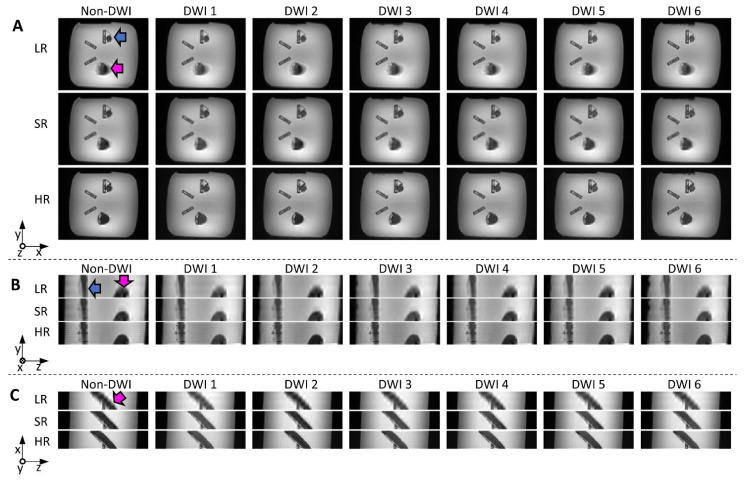
Reconstructed images from the physical helical phantom shown in x-y (**A**); y-z (**B**); and x-z (**C**) planes (non-DWI and DWI from six diffusion directions): low-resolution (LR) and super-resolution (SR) were obtained with the same scan time (3.5 min); native high-resolution images (HR, 14 min scan time) are shown for reference. The pink and blue arrows indicate the straight and helical bundles, respectively. Blurring can be seen on LR images, especially in the x-z plane (bottom views).

Reconstructed images from the physical phantom are shown in Figure 6. The LR (transverse orientation) and SR images required the same scan time (3.5 min, 6 diffusion directions × 5 Nex × 3 in both cases), while the HR images (6 directions, 60 Nex) took 14 min. In the LR images, the edges of the helical bundle appear blurred (due to the 8 mm thickness and oblique orientation of the bundle), especially in the x-z plane, where edges are clearly less sharp than for other methods. SR images visually seem close to the HR reference, with little blurring, and do not appear noisy, despite the short scan time compared to HR. Quantitative SNR measurements are included in Table 1 and confirm the visual inspection, with the highest SNR found in SR images.

**Figure 7:**
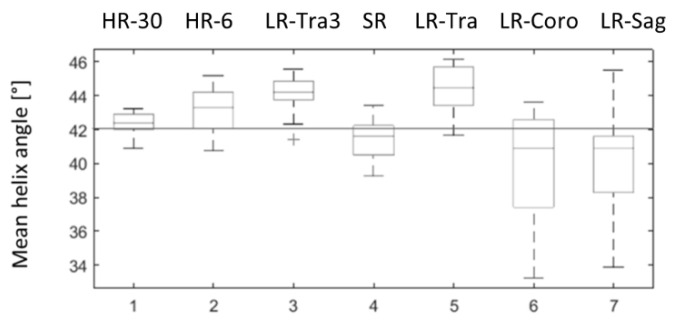
Distributions of mean helix angle measured in the physical phantom over 20 ROI positioned at different levels along the z axis. 1: ground truth HR with 30 diffusion directions (HR-30); 2: HR with six diffusion directions (HR-6); 3: LR in transverse orientation and three repetitions (LR-Tra3); 4: SR reconstruction (SR); 5, 6, 7: transversal (LR-Tra), coronal (LR-Coro) and sagittal (LR-Sag) acquisitions used as inputs for SR reconstruction.

HA measurements in the physical phantom were overestimated compared to the theoretical value for all acquisitions in the transverse orientation and were underestimated in coronal and sagittal orientation, as shown in Figure 7. HA measurements obtained with SR were closer to the ground truth (42°) than the low-resolution images, both in terms of accuracy (mean) and precision (standard deviation): HA was 41.53° ± 1.10° with SR, 44.34° ± 1.34° with LR in the transverse orientation (LR-Tra), 39.93° ± 3.16° with LR in the coronal orientation (LR-Coro), and 40.28° ± 2.8° with LR in the sagittal orientation (LR-Sag). The transverse LR image with higher SNR (LR-Tra3, Nex = 3) showed biased HA values, as for LR-Tra, and a standard deviation similar to that of SR: 44.07° ± 1.06°. However, SR reconstruction was less biased in comparison to the theoretical value than LR acquisition. SR measurements of HA were also closer to HR measurements with 30 diffusion directions. More detailed statistics for each of the datasets are presented in Table 1. In terms of the classic diffusion metrics, MD and FA obtained with SR were more consistent with HR using the same 6-direction dual gradient scheme, although FA was slightly overestimated with HR-6 (6 directions) compared to the HR-30 reference (30 directions). Finally, TA values show a lower variance with methods using a thick transverse plane (LR-Tra3, LR-Tra), as in the numerical simulation.

### 3.3. In Vivo Data

**Figure 8:**
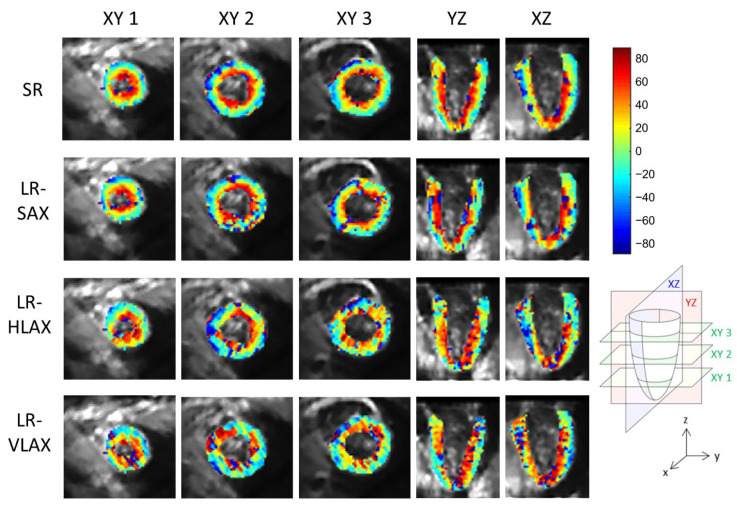
Example of in vivo HA maps superposed to non-diffusion-weighted magnitude images and reformatted to XY, YZ and XZ view (these strictly orthogonal views are defined in the schema on the right). From top to bottom, super-resolution reconstruction (SR), low-resolution volume acquired in short-axis (LR-SAX), horizontal long-axis (LR-HLAX) and vertical long-axis (LR-VLAX) orientation. All images were interpolated to 1 × 1 × 1 mm^3^ for display.

**Figure 9:**
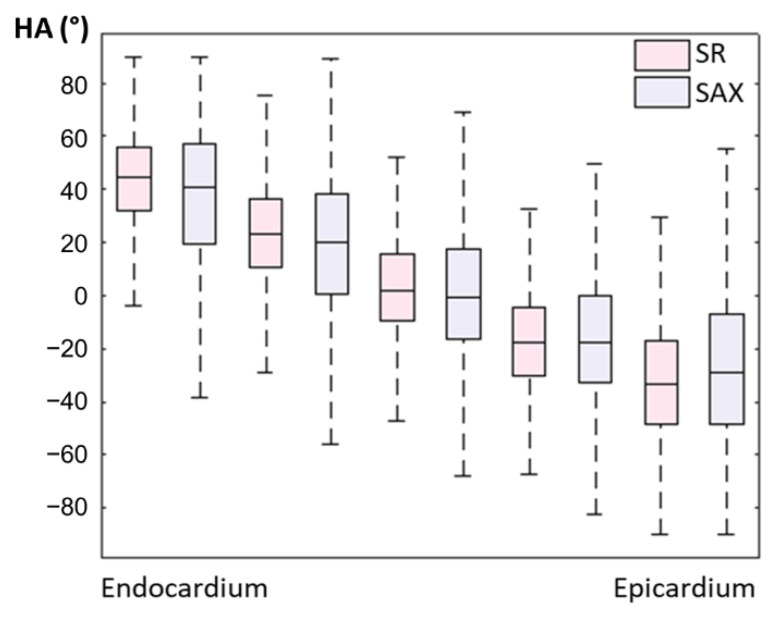
Evolution of in vivo HA measurement from endocardium to epicardium for SR and LR SAX volumes (pooled data from the whole LV and from N = 6 subjects).

An example of a full DWI dataset showing reconstructed SR and SAX LR (after interpolation) is provided as Supplementary Video S4. In vivo HA maps, reformatted to XY, YZ and XZ orientations, are shown in Figure 8. The helicoidal organization of myofibers is well described in both SR and SAX images at the mid-cavity level. Nonetheless, at the apical level a loss of linearity in the transmural HA profile (from endocardium to epicardium) occurs for SAX orientation. With the LR HLAX acquisition, a gradual change of the helix angle from positive to negative values can only be seen in a portion of the LV wall. The structure does not appear clearly in any view of the LR VLAX acquisition. Figure 9 shows the quantitatively measured transmural profile of HA for SR and SAX volumes, estimated globally for the LV. The interquartile range is smaller for SR reconstruction and there is less overlapping between layers. The variance in each layer is relatively large since the data are pooled from the whole LV (different myocardial segments and slices from base to apex) and from the six subjects, reflecting the intersegment and intersubject variability. The changes in HA as a function of distance to endocardium, in percentages of the wall thickness, as well as TA, MD and FA measurements for each dataset are summarized in Table 2. Detailed statistics for each volunteer can be found in the Supplementary Materials Table S2. When comparing HA transmural profiles obtained from the three LR datasets, SAX provides the steepest slope and the lowest variance, as expected. The slope obtained from the SR data (−0.993 ± 0.007) is higher than that from the LR SAX data (−0.873 ± 0.010), and its variance is also smaller. MD values differ between the LR datasets with different orientations. The SR values of MD (1.35 ± 0.28) provide intermediate values with a lower variance (between 1.30 ± 0.42 and 1.50 ± 0.43 for LR data). FA values are lower with SR (0.35 ± 0.14) than with LR data (0.46  ± 0.19 for SAX), likely because SR data had better SNR. The same observation can be made for TA.

**Figure 10:**
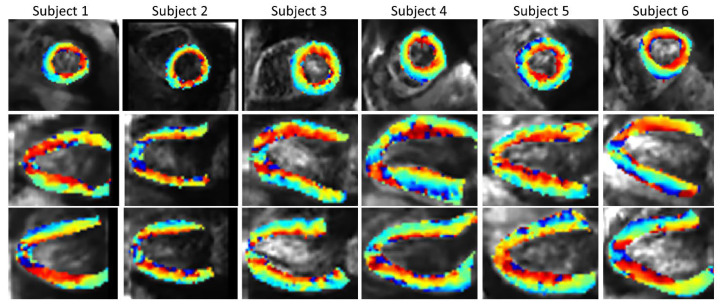
In vivo HA maps from all six subjects (2 × 2 × 2 mm^3^ interpolated to 1 × 1 × 1 mm^3^ for display), superposed to non-diffusion-weighted magnitude images and reformatted to XY, YZ and XZ view.

Figure 10 shows the SR HA maps obtained from all six subjects. The overall SNR gain with the SR reconstruction across the volunteers (Supplementary Materials Table S3) is 1.88 in comparison to SAX volume, 1.51 in comparison to HLAX volume and 1.89 in comparison to VLAX volume.

## 4. Discussion

As previously shown in [31,32], the SR method allows, with an equivalent acquisition time, the improvement of both the spatial resolution and SNR. In our numerical phantom, although noise caused large errors in the HA estimates with a native HR image, errors were evenly distributed across the volume and no bias was observed. In the case of LR SAX images, errors are minimal when the direction of the first eigenvector varies slowly along the z axis. When the curvature is steeper, and changes are more abrupt, a stair-casing effect appears on both the magnitude images and HA maps. In this configuration, errors become significant. Overall, the SR images provided slightly higher errors in the HA maps than LR SAX images (with three repetitions) in our numerical phantom. This can be explained by the geometry of the heart which favors the reconstruction of structures with marked changes within the imaging plane and moderate changes in the through-plane direction. Therefore, in the mid-cavity and in the base of the LV, the higher SNR of the LR images prevailed over SR. The SR method is also affected by errors that come from stacks in YZ and XZ orientation. However, SR did provide more robust results in detecting small changes near the apex, where the curvature of the LV surface is highest. Overall, the distribution of errors across the volume was more uniform with SR because of the combination of the three orientations that mitigate and distribute the error over all directions.

These results were confirmed with the physical helicoidal phantom. Coronal and sagittal scans showed larger errors in HA measurements than the transverse scan. Indeed, in this phantom, there was no change of the eigenvector direction in the transverse direction. Nevertheless, all LR acquisitions led to a relatively large bias in HA estimates, with an overestimation for the coronal and sagittal scans and an underestimation for the transverse scans. Several factors could explain this result, including the use of the dual gradient scheme (with only six directions, non-uniformly distributed on a sphere) which may lead to an anisotropic noise propagation [34], and imperfections/asymmetries of the gradient coil system, which both may lead to orientation-dependent measurements [35,36,37]. The bias was significantly mitigated with the SR strategy, and SR estimates were close to the HR DTI scan with 30 diffusion directions. Therefore, the combination of three scans with different orientations may reduce the dependence of the measurement on the orientation and the underlying structure.

Considering the special geometry of the heart and the organization of cardiomyocytes, an SAX acquisition at the mid-ventricular level is the optimal way to acquire DWI. Nevertheless, this assumes that there are no marked changes across the slab. In vivo measurements are consistent with the results in the phantoms. Indeed, in the SAX LR images the changes of HA across the myocardium appear similar to those reported in the literature at the basal and mid-ventricular levels; however, this structure is not well preserved at the apical level. The SR strategy reduces the global errors on diffusion metrics and provides a better assessment of HA changes across the wall as shown in Table 2. It is difficult to conclude on bias of our in vivo measurements with the different methods because no ground truth is available. In particular MD and FA values reported in the literature are dependent on the sequence used to acquire the data [17,38]. Nevertheless, the HA gradient across the wall, MD and FA mean values are consistent with the literature [38]. A benefit of the SR strategy is to improve the global image quality that permits a better visualization of the underlying structure in the three orthogonal orientations and improves SNR at the same time as resolution. Isotropic voxels, which are better suited for tractography algorithms, can be reached in a scan time equivalent to that of previous studies targeting a whole heart coverage [10,18,19].

An important challenge for applying the proposed SR strategy to in vivo cDTI is the motion correction step, which is needed to ensure consistency of all input data and prevent motion blurring. The proposed approach allows the definition of a self-consistency metric based on all of the slice intersections in the dataset. If only a single orientation was acquired, registration to a separate 3D anatomical scan would be possible, but such a slice-to-volume registration with data from different sequences (with different imaging contrast) would be even more challenging. Our method has some limitations though that should be mentioned. Only rigid transformations of the heart region can be handled. The optimization problem remains challenging because the heart has a high degree of symmetry, which can lead to several local minima, and it may be sensitive to various imaging artifacts. The global convergence of the optimization routine is not proven, although in our datasets the parameters did not change much after the first iterations.

Another limitation of the study is that it is a feasibility study on healthy subjects. The method should also be applied to patients with various cardiomyopathies before its potential benefit can be fully assessed. 

Finally, a possible improvement would be to apply this technique with an echo navigator to manage breathing motion. If the navigator was perfectly reliable, the super-resolution algorithm would not need post-processing motion correction. In practice, especially with a lengthy acquisition, there may be a drift of the organs which could be corrected with the proposed post-processing method. The SR strategy itself should also be applicable to other cDTI acquisition techniques, including both spin-echo and STEAM sequence families [39,40]. Indeed, the improvement brought by the SR strategy, compared to conventional short-axis-only scans, is independent of the protocol parameters, such as the number of diffusion directions, the number of averages, the choice of the cardiac phase (mid-systole or diastole).

## 5. Conclusions

In conclusion, the study shows the feasibility of super-resolution using three (nearly) orthogonal slice stacks for assessing myocardial architecture by cDTI. In vivo application was rendered possible by a motion correction step ensuring self-consistency of all slice intersections in the dataset. This strategy was shown to provide a good trade-off between scan time and accuracy of cDTI metrics, including MD, FA, HA and TA.

## Figures and Tables

**Table 1:** Signal-to-noise ratio, mean diffusivity, fractional anisotropy, minimal HA, maximal HA and average HA over ROIs in the physical phantom (ROIs drawn in the helical shaped bundle, across 20 slices).

**Dataset**	**SNR**	MD $\left(10^{-3}{\mathrm{mm}}^{2}/s\right)$	FA	HA Mean (°)	HA Min (°)	HA Max (°)	TA Mean (°)
GT		-	-	42.07	34.57	49.57	0
HR 30dir	70.8	1.69 ± 0.11	0.31 ± 0.06	42.40 ± 0.56	33.71 ± 5.51	49.64 ± 1.70	−2.03 ± 3.35
HR	64.8	1.71 ± 0.09	0.34 ± 0.07	43.21 ± 1.26	31.76 ± 4.10	52.19 ± 3.05	−0.15 ± 2.67
LR-Tra3	68.8	1.72 ± 0.21	0.32 ± 0.14	44.07 ± 1.06	36.96 ± 2.15	52.05 ± 2.45	−0.97 ± 1.98
SR	81.5	1.68 ± 0.12	0.35 ± 0.09	41.53 ± 1.10	31.90 ± 3.31	49.48 ± 2.36	−1.17 ± 2.34
LR-Tra	67.4	1.73 ± 0.21	0.32 ± 0.14	44.34 ± 1.34	34.75 ± 1.83	52.28 ± 2.38	−1.34 ± 1.89
LR-Coro	76.8	1.72 ± 0.24	0.31 ± 0.15	39.93 ± 3.16	26.81 ± 7.13	49.46 ± 2.76	−0.25 ± 3.11
LR-Sag	75.7	1.72 ± 0.20	0.35 ± 0.14	40.28 ± 2.85	28.01 ± 4.59	52.71 ± 3.84	−1.72 ± 3.57

**Table 2:** In vivo diffusion metrics over the left ventricular myocardium for SR, SAX, HLAX and VLAX (pooled data from the whole LV and from N = 6 subjects).

Dataset	HA Slope (°/%)	TA (°)	$\mathrm{MD}\;(10^{-3}{\mathrm{mm}}^{2}/s)$	FA
SR	−0.993 ± 0.007	−2.22 ± 24.13	1.35 ± 0.28	0.35 ± 0.14
SAX	−0.873 ± 0.010	−0.15 ± 32.39	1.50 ± 0.43	0.46 ± 0.19
HLAX	−0.643 ± 0.013	−2.80 ± 32.81	1.40 ± 0.43	0.42 ± 0.18
VLAX	−0.635 ± 0.013	−4.04 ± 31.52	1.30 ± 0.42	0.45 ± 0.20

## Data Availability

Not applicable.

## Supplementary Materials

The following are available online at https://www.mdpi.com/article/10.3390/diagnostics12040877/s1, Table S1: Comparison to ground truth (GT) of mean diffusivity (MD), fractional anisotropy (FA) and transverse angle (TA) derived from the simulated HR, LR and SR DWI in the numerical phantom; Table S2: Slope of the evolution of helix angle (°/%) as a function of the distance to endocardium in percent for each healthy volunteer; Table S3: SNR gain of SR reconstruction with respect to the native images (SAX, HLAX, VLAX); Video S4: Slice-by-slice view of a subject dataset, including SR and native SAX images (b_0_ image and the 6 DW images).
