# Supplementary material for: In Vivo Super-Resolution Cardiac Diffusion Tensor MRI: A Feasibility Study
Source: Diagnostics (Basel). 2022 Mar 31;12(4):877. doi: 10.3390/diagnostics12040877 (PMC9028988; doi:10.3390/diagnostics12040877)
Supplement: Supplementary file 1 [file diagnostics-12-00877-s001.zip › SupplementaryMaterial Tables S1-S3.pdf]

## SUPPORTING TABLES

**Table S1.** Comparison to ground truth (GT) of mean diffusivity (MD), fractional anisotropy (FA) and transverse angle (TA) derived from the simulated HR, LR and SR DWI in the numerical phantom

| Dataset | MD ($10^{-3}$ mm²/s) | FA | TA (°) |
|---|---|---|---|
| GT | 1.5 | 0.32 | 0 |
| HR | 1.50 ± 0.06 | 0.33 ± 0.05 | -0.05 ± 8.15 |
| LR | 1.45 ± 0.02 | 0.31 ± 0.02 | -0.07 ± 1.81 |
| SR | 1.50 ± 0.02 | 0.31 ± 0.02 | 0.03 ± 2.11 |

**Table S2.** Slope of the evolution of helix angle (°/%) as a function of the distance to endocardium in percent for each healthy volunteer

| | SR | SAX | HLAX | VLAX |
|---|---|---|---|---|
| 1 | -0.966 ± 0.021 | -0.794 ± 0.037 | -0.839 ± 0.029 | -0.673 ± 0.040 |
| 2 | -1.036 ± 0.020 | -0.937 ± 0.027 | -0.745 ± 0.040 | -0.611 ± 0.034 |
| 3* | -0.955 ± 0.015 | -0.832 ± 0.021 | -0.864 ± 0.022 | -0.695 ± 0.025 |
| 4 | -0.971 ± 0.014 | -1.033 ± 0.028 | -0.565 ± 0.028 | -0.740 ± 0.023 |
| 5 | -0.983 ± 0.015 | -0.962 ± 0.022 | -0.714 ± 0.024 | -0.774 ± 0.027 |
| 6 | -1.057 ± 0.018 | -0.573 ± 0.031 | -0.234 ± 0.039 | -0.30 ± 0.039 |

* *acquisition with a b-value of 500 s/mm²*

**Table S3.** SNR gain of SR reconstruction with respect to the native images (SAX, HLAX, VLAX)

| | SAX | HLAX | VLAX |
|---|---|---|---|
| 1 | 1.94 | 1.09 | 1.14 |
| 2 | 2.19 | 1.05 | 1.17 |
| 3 | 1.40 | 1.55 | 1.92 |
| 4 | 2.04 | 1.60 | 2.52 |

| | | | |
|---|---|---|---|
| 5 | 1.58 | 1.44 | 1.62 |
| 6 | 2.16 | 2.35 | 2.98 |
| **Mean** | **1.88** | **1.51** | **1.89** |
